# Sex estimation standards for medieval and contemporary Croats

**DOI:** 10.3325/cmj.2017.58.222

**Published:** 2017-06

**Authors:** Željana Bašić, Ivana Kružić, Ivan Jerković, Deny Anđelinović, Šimun Anđelinović

**Affiliations:** 1 University Department of Forensic Sciences, University of Split, Split, Croatia; 2 University Hospital Center Split, Department of Dermatovenerology, Split, Croatia; 3 University of Split, Rector’s Office, Split, Croatia

## Abstract

**Aim:**

To develop discriminant functions for sex estimation on medieval Croatian population and test their application on contemporary Croatian population.

**Methods:**

From a total of 519 skeletons, we chose 84 adult excellently preserved skeletons free of antemortem and postmortem changes and took all standard measurements. Sex was estimated/determined using standard anthropological procedures and ancient DNA (amelogenin analysis) where pelvis was insufficiently preserved or where sex morphological indicators were not consistent. We explored which measurements showed sexual dimorphism and used them for developing univariate and multivariate discriminant functions for sex estimation. We included only those functions that reached accuracy rate ≥80%. We tested the applicability of developed functions on modern Croatian sample (n = 37).

**Results:**

From 69 standard skeletal measurements used in this study, 56 of them showed statistically significant sexual dimorphism (74.7%). We developed five univariate discriminant functions with classification rate 80.6%-85.2% and seven multivariate discriminant functions with an accuracy rate of 81.8%-93.0%. When tested on the modern population functions showed classification rates 74.1%-100%, and ten of them reached aimed accuracy rate. Females showed higher classification rates in the medieval populations, whereas males were better classified in the modern populations.

**Conclusion:**

Developed discriminant functions are sufficiently accurate for reliable sex estimation in both medieval Croatian population and modern Croatian samples and may be used in forensic settings. The methodological issues that emerged regarding the importance of considering external factors in development and application of discriminant functions for sex estimation should be further explored.

Sex estimation is one of the first steps of biological or forensic anthropological analysis. The main reason for this is the fact that other vital information, such as age and stature, cannot be adequately obtained without initial sex estimation (1-6). Also, accurate sex estimation is essential in all of the steps of the identification process, beginning with narrowing down the list of individuals, enabling identification, to drawing the final conclusions.

There are three methodological approaches to reveal the sex of skeletal remains: the analysis of DNA, analysis of morphological traits that exhibit sexual dimorphism, and osteometric methods. The analysis of DNA is the gold standard and indisputably the most accurate method for sex determination. Nevertheless, it is also a lengthy and very expensive procedure that can be obstructed by insufficient bone preservation, amount of extracted DNA, and inhibitors (7). Morphological methods are based on the analysis of skeletal features showing pronounced sexual dimorphism, mainly concentrated on the pelvis where clear distinctions of shape and bone configurations are macroscopically detectable. Sex obtained by morphological methods can at best be considered as “assessed”, because morphological methods with the statistical background (8,9) are rarely used. Thus, the main component of estimation method, ie, data on the accuracy of implemented methodology, in most cases remains unknown.

If the pelvis is not adequately preserved and the skeletal remains are fragmented, sex can be anthropologically estimated only using osteometric methods. That includes various statistical approaches, such as discriminant function analysis or logistic regression, which classify skeletal remains of unknown individuals into one of two categories (female or male) by single or multiple measurements of one or more bones (10). Its main advantage is a reduction of subjectivity and the availability of data on the accuracy of each method which is an indispensable requirement in a forensic environment.

One of the widely accepted methods of osteometric sex estimation is the discriminant function analysis; only recently have two studies included all standard skeletal measurements (11,12). Spradley and Jantz (11) developed univariate functions for 78 standard measurements (13), and showed that sometimes single long bone measurement could provide better classification rate than the multivariate analysis of the cranium, which had been long considered as an excellent sex indicator (11). However, the limitation of discriminant functions is their population specificity; therefore, they should be developed for each population or region separately. Some studies have also shown that discriminant functions designed for anthropological samples can be applied to contemporary samples from the same area (14).

Although several studies have been carried out on medieval and contemporary Croats (15-20), only one analyzed the complete skeleton and explored the best combination of skeletal measurements for sex estimation (12). For this reason, the primary aim of this study was to develop the first sex estimation standards on the whole skeleton for medieval Croatian population. The second aim was to test if the developed functions are applicable to modern Croatian population.

## MATERIAL AND METHODS

### Material

We analyzed a sample of 519 skeletal remains from 10 medieval Eastern Adriatic coast sites including Ostrovica Greblje (9th century) (21), Svećurje–Žestinj (9/11th century) (22), Rižinice (9/10th century), Bijaći – Stombrate (9/10th century) (23), Sv. Mihovil – Kučiće (12th-14th century) (24), Šopot – Benkovac (14/15th century) (25), Kamen Most – Kaldrma (14/15th century) (26), Gornji Koljani – Crkvina (14th-16th century) (27), Plina (14th-16th century) (28), and Otok Vuletina rupa – Grebčine (17/18th century) (29). From the sample of 519 skeletons, for purposes of this research, we chose 84 adult persons with preserved more than 80% of bones enlisted in standard measurements (30), and free of premortem or postmortem changes that could affect the measurements (31).

To compare these results to the contemporary population, we additionally used two contemporary skeletal samples. The first sample consisted of 19 female skeletons from the Kozala monastery graveyard (Rijeka, Croatia) from the 19/20th century (7,12). The second sample encompassed skeletal remains of the victims of World War II from site Dubrava, consisting of 17 males and one female.

### Methods

In cases of preserved pelvis, we assessed sex by the examination of sex-specific morphological characteristics including the greater sciatic notch, ventral arc, subpubic concavity, medial aspect of ischiopubic ramus, and preauricular sulcus (12,32-34). In cases where pelvis was not preserved or morphological indicators were not consistent, we conducted an analysis of ancient DNA, ie, amelogenin locus using previously described protocol (12,35-37).

We measured 75 standard skeletal measurements (30). As a mandibulometer was not available, we did not take three mandibular measurements (12).

We measured both left and right bones and tested if there were differences between left and right measurements (12). For those measurements that showed statistically significant differences between left and right bones, we included only left bones in the analysis (data not shown). Otherwise, in cases of missing values of left bones, we replaced them with right bone measurements. As the sample spanned a broad range of time (8th-17th century), we also tested if the bone measurements showed secular changes. At that end, the sample was divided using archaeological data in two groups. The first group consisted skeletons from Early Middle Ages (8th-12th century), whereas the second group consisted of skeletons from Late and High Middle Ages (12-17th century) (12).

### Statistical analysis

We calculated means and standard deviations for all measurements and using univariate ANOVA tested if they show statistically significant sexual dimorphism. We included in the further study only those measurements that did show significant differences. We calculated univariate sectioning points for all measurements and calculated multivariate discriminant functions for all bones. Accuracy (ie, classification rates) of functions were assessed using the jack-knife method or cross-validation. This procedure calculates each function by leaving out one of the cases, in turn, calculating the function based on the remaining cases, and then classifying the left-out case. We computed sectioning points and discriminant functions for numerous combinations, but we included in the research only those functions with classification rate above 80%. We performed statistical analysis using SPSS (version 17, SPSS Inc., Chicago, IL, SAD), with a statistical significance set at *P* < 0.01.

## RESULTS

For 49 individuals sex was successfully estimated by examination of pelvic features, and for 35 individuals the analysis of the DNA was performed. Although not all full nuclear DNA profiles were gained (8 full profiles and 27 partial), amelogenin locus was successfully amplified in all cases. In the end, total sample consisted of 41 female and 43 male (12).

In the first step of the analysis, we excluded pelvic bones and scapulae as they were not represented in the sufficient number to perform the analysis. Due to secular changes, we excluded only bizygomatic breadth for further analysis as it showed statistically significant difference between of males from two periods (*P* = 0.002) (12).

Only ten cranial measurements showed statistically significant sexual dimorphism (*P* < 0.01). Therefore, we included only those in further analysis (maximum length, maximum breadth, cranial base length, maxilla, alveolar breadth, upper facial height, minimum frontal breadth, upper facial breadth, biorbital breadth, interorbital breadth, frontal chord, and parietal chord). On the mandible, the only measurement that showed sexual dimorphism was mandibular body height. Almost all included postcranial bone measurements showed statistically significant sexual dimorphism. The measurements that we excluded from the further analysis were femoral AP subtrochanteric diameter, tibial transverse diameter at nutrient foramen and all measurements of the sacrum, as they did not show significant sexual dimorphism (*P* > 0.01) (12).

From those variables that showed sexual dimorphism, we computed univariate discriminant functions, but we included only five single bone measurements that met accuracy criteria of 80% (Table 1). We constructed discriminant functions for all bones and included only those that met the criteria. It was possible for femur, ulna, humerus, clavicle, calcaneus, tibia, and cranium (Table 2). In the next step, we tested booth univariate and multivariate functions on modern Croat samples and showed that most of them could be applied to the modern sample (Table 3).

**Table 1:** Single bone measurements with highest classification accuracy shown by descriptive statistics, demarking points, ANOVA results, and accuracy rates

Measure/measurement number (30)	Females		Males		*P*	Demarking point	Females (n/N)	Males N (n/N)	Overall (n/N)	Overall accuracy (%)
	n	mean±SD*	n	mean±SD						
Ulna: physiological length / 51	28	220.2 ± 12.3	33	244.8 ± 15.3	<0.001	232.5	26/28	26/33	52/61	85.2
Femur: maximum head diameter / 63	33	42.9 ± 3.1	38	48.9 ± 3.4	<0.001	45.9	29/33	31/38	60/71	84.5
Humerus: epicondylar breadth /41	35	55.1 ± 4.8	34	64.1 ± 4.4	<0.001	59.6	32/35	26/34	58/69	84.1
Calcaneus: middle breadth /78	22	38.6 ± 2.4	28	42.9 ± 2.9	<0.001	40.8	19/22	23/28	42/50	84.0
Ulna: maximum length /48	27	250.2 ± 14.5	35	272.2 ± 15.6	<0.001	261.2	22/27	28/35	50/62	80.6

*Standard deviation.

**Table 2:** Multivariate discriminant functions and classification rates for femora, ulnae, humeri, clavicle, calcanei, tibiae and crania

Bone	Measurements	Sectioning point	Females (n/N)	Males (n/N)	Overall (n/N)	Overall accuracy (%)
Femur	bicondylar length x 0.011 + maximum head diameter x 0.282 + circumference at midshaft x 0.052 - 22.782	-0.074	25/27	28/30	53/57	93.0
Ulna	maximum length x (-0.004) + physiological length x 0.068 + minimum circumference x 0.219 - 23.725	-0.157	23/24	27/32	50/56	89.3
Humerus	epicondylar breadth x 0.186 + head diameter x 0.221+ maximum diameter at midshaft x (-0.085) -18.987	-0.093	23/25	24/29	47/54	87.0
Clavicle	maximum length x 0067 + sagittal diameter x 0.439 + vertical diameter x 0.151 - 16.156	-0.046	26/29	27/32	53/61	86.9
Calcaneus	middle breadth x 0.315 + maximum length x 0.073 - 18.998	-0.194	19/22	23/27	42/49	85.7
Tibia	length x 0.024 + proximal epiphyseal breadth. x 0.143 + circumfernce at nutrient foramen x 0.016 - 20.871	-0.074	23/26	23/30	46/56	82.0
Cranium	base length x 0.078 + minimum frontal breadth x 0.191 + maximum alveolar breadth x 0.035 - 28.832	-0.078	22/25	23/30	45/55	81.8

**Table 3:** Validation of univariate and multivariate discriminant functions for sex estimation on the modern sample (20 females and 17 males)

Function / Measure	Females (n/N)	Males (n/N)	Overall (n/N)
Ulna: physiological length / 51	16/17	4/4	20/21
Femur: maximum head diameter / 63	18/19	17/17	35/36
Humerus: epicondylar breadth / 41	20/20	10/11	20/21
Calcaneus: middle breadth / 78	12/17	8/10	20/27
Ulna: maximum length / 48	17/17	3/3	20/20
Femur	17/19	13/13	30/32
Ulna	15/17	3/3	18/20
Humerus	19/20	7/7	26/27
Clavicle	14/17	6/6	20/23
Calcaneus	14/17	8/9	22/26
Tibia	16/18	10/12	26/30
Cranium	11/13	-	-

## DISCUSSION

This is the first study that comprised all standard measurements and tested their application for the sex estimation in one ancient population and afterward on the modern sample consisting of females and males. This study examined sexual dimorphism in Medieval Croatian population providing seven multivariate and five univariate discriminant functions for sex estimation with overall accuracy rates above 80%. The results also showed a great potential for the application of the developed standards on the contemporary population, ie, on forensic sample. Also, it is important to emphasize that this is one of the fewer research that used analysis of aDNA for sex determination which makes “input data” about sex as reliable as those from modern skeletal collections, thus enabling application of discriminant functions in forensic cases.

We included only sufficiently preserved skeletons, took almost all standard measurements and tested if the measurements show sexual dimorphism. Unfortunately, not all measurements could be included in the study. The first reason was the insufficient preservation of scapulae and pelvic bones. However, this deficiency does not represent a significant shortage to the study as scapula is fragile (38) and is rarely completely preserved in archaeological and forensic context. This issue could probably be overcome by the implementation of new measurements which are already included in the updated version of standard measurements (39). The lack of pelvic measurements also does not represent a substantial drawback. Specifically, a majority of those measurements according to recommendations in new standards (39) are replaced with new ones, because of unacceptable measuring errors and unclear definitions of measurements (40). After the non-population specific method for probabilistic sex diagnosis on pelvic bones (41) has been developed and validated (42-44), providing minimum accuracy in original study of 98.7% and 100% in validation studies, there is no more need for developing novel discriminant function for pelvic bone.

The second reason for exclusion of some of the measurements was the lack of statistically significant sexual dimorphism. It was most notable for cranial measurements as only ten of them showed statistically significant sexual dimorphism. These results additionally confirmed the findings of Spradley and Jantz (45) and reaffirmed that cranium is not such a good sex indicator as it was previously considered. Postcranial measurements that lacked sexual dimorphism were femoral AP subtrochanteric diameter and tibial transverse diameter at nutrient foramen, which is not often the case in these types of studies (45-47). Nonetheless, this finding could be explained by the position of muscular attachments near the measuring landmarks that are a consequence of heavy physical labor which in medieval times was common both for man and woman, thus lowering the degree of sexual dimorphism. Regarding the sacrum, the lack of sexual dimorphism is not surprising finding as it was also common in other studies (10,12,48).

We also tested the sample homogeneity and possible occurrence of secular changes. As we excluded only one measurement we concluded that the sample is representative for the whole period of the Middle Ages (12).

In the present study, we developed univariate discriminant functions for all available measurements that showed statistically significant sexual dimorphism and computed multivariate discriminant functions for each bone. The primary criterion was accuracy rate of 80% which should be a minimum requirement for sex estimation in a practical sense (49) generally in anthropology, but especially iin forensic settings.

The results of the study showed that five bones had overall accuracy rate 82%-93% when multivariate analysis was performed. In the study of Spradley and Jantz (45), the same bones also had high classification rates, but no significant regularities were found in order of accuracy rates. It is possible to highlight accuracy rate and applicability of calcaneal measurements. This find can be very useful in forensic context, as from our experience calcaneus is usually well preserved probably due to its size and built which prevents deterioration that often occurs in longer or thicker bones.

For univariate discriminant functions, only five bone measurements showed accuracy above 80% and four of them (Ulna Physiological Length/51, Femoral Maximum Head Diameter/63, Humerus Epicondylar Breadth/41, Calcaneus Middle Breadth/78), reached higher classification rates than multivariate discriminant functions of tibia and cranium. The same measurements are also of the greatest importance in practical application as they do not require a bone to be complete.

Generally speaking, in comparison with the study of Spreadly and Jantz (45), our study showed a lower degree of sexual dimorphism. In the named study, there were 11 and 12 discriminant functions (including bones that were not sufficiently preserved in our study) that performed above 80% for the black and white population when using multivariate approach. Also, there were 18 and 19 single bone measurements that showed classification rates higher than 80%. On the other hand, in our study, there were seven multivariate and only five univariate discriminant functions that met established criteria.

As discriminant functions are usually population-specific, the majority of differences from the study above can be simply explained by different population affinity but also by different life-conditions and occupation. However, the same deviation is also visible when comparing the results to the available standards for the modern Croatian population for single bones (46,47).

In this regard, it is important to consider the historical period and associated environmental factors. Namely, during the Middle Ages people were subdued to heavy physical labor and physiological stress stemming out from poor quality of life and malnutrition (50), and the analyzed population showed similar frequencies of life quality markers for males and females in previous study (51). Physical labor could thus influence the sexual dimorphism as functional demands of weight bearing and muscle activity increase the bone dimensions (52). Therefore, the exposure of woman to heavy physical labor could reduce such differences between males and females. It is also important to highlight that environmental factors such as the malnutrition and disease can cause the growth stunting. However, as woman are more resistant to these factors, the sexual dimorphism in body size can additionally be reduced. The reason for this is still unknown, but we can assume that main reasons are pregnancy and childbirth. Namely, females are less prone to the influence of environmental stressors that enables the species to survive (53).

As expected, the cranium performed worse than six multivariate and four univariate discriminant functions reaching accuracy rate of 81.8%. However, the cranium performed better than multivariate discriminant functions for radius, fibula, mandible, sacrum and all remaining univariate discriminant functions, but also had a better degree of preservation than pelvic bones and scapulae. One advantage the cranium in populations in which sexual dimorphism was in some way compromised could be the lower influence of environmental functions. Although it is not entirely resistant to them, and certain changes reflect even on the cranial shape (9), it is probably less prone to those factors and probably won’t be to that extent affected like long bones. Therefore, cranium should not be considered as such useless sex indicator as it was previously presented (11).

Despite the less pronounced sexual dimorphism, developed discriminant functions enabled a reliable sex estimation both in the medieval sample that was used for calculation of the functions as well as in the modern skeletal sample. Only one function - calcaneus middle breath with a classification accuracy of 74.1% did not perform well, while accuracy rates for other functions were between 84.6 and 100%. All the functions except those for calcaneus reached even higher accuracy on the modern sample than on the original. Until now, for modern Croatian population, only discriminant functions for femora and tibiae have been developed (46,47). In a practical sense, the most important results are discriminant functions for clavicles, humeri, ulnae, and calcanei. Namely, standards for sex estimation on the modern population using these bones have not still been developed, and they performed sufficiently well to be employed in the forensic cases. Unfortunately, discriminant functions for crania could not be completely tested on the modern population as the bones in the male sample were not adequately preserved to conduct the analysis. However, even though it achieved relatively high classification accuracy for females (85%), it is not at that extent important for sex estimation as it is significant for population affinity. This topic has been covered in the previous study that showed a strong association between genetic markup and cranial measurements (37). Thus, it additionally supports the application of developed functions on the modern sample.

It is not surprising that discriminant functions developed for one sample may apply to samples from other periods but the same geographical area (7,12,14) or that they reach higher accuracies on test sample like in our case. This phenomenon is most likely a consequence of the same or the similar origin, similar morphology, and similarly expressed sexual dimorphism (14). Higher classification rates also can be explained. Although at first, this finding does not make sense, the answer most likely also lies in the lower degree of sexual dimorphism as it was elaborated previously. The main reason for this is the fact that the original sample originates from the medieval period that is generally characterized by the lower quality of life and more intensive physical labor, whereas in the modern sample living conditions and life expectancy have considerably improved (54). It is well visible if we compare classification rates in males and females in the medieval and modern sample and consider that females are less prone to environmental factors. Namely, in almost all functions in medieval sample females reached somewhat higher accuracy rates than males, whereas in the modern sample in the majority of cases males reached higher classification rates than females. Accuracy rates have also increased for females but not to that extent as it was for males. For example, for maximum head diameter accuracy rate in females increased from 87.9% (29/33) to 94.7% (18/19) and for males from 81.6% (29/33) to 100% (17/17). Despite the fact that the classification rates showed differences, it does not, in any case, mean that those functions are less applicable. In this regard, it is also important to stress that average heights did not substantially increase until the early 20th century and that heights that are common today were reached in the second half of the 20th century (54). Therefore, the functions developed in our study could be even more applicable to populations from 19th and the first half of the 20th century, what is of particular importance for investigation of mass graves from WWII.

Although it was not the primary goal of the research, this study has drawn attention to several important forensic issues in sex estimation. First, environmental factors that may influence sexual dimorphism in particular historical periods should, when available, always be considered to confirm that referent skeletal collection does represent the modern population. The study also showed that the cranium, even though it is not the best sex indicator, can be very useful for sex estimation in populations where long bones show a lesser degree of sexual dimorphism. Also, it can be used to confirm population homogeneity when we are testing discriminant functions on populations from the other periods.

Furthermore, the study showed an advantage of implementing aDNA analysis for sex determination that is rarely applied both in ancient and forensic samples. In this context, when using aDNA analysis, we can overcome the lack of historical or forensic data on skeletal remains, and thus convert the unknown collection to referent collection which would in future be used for developing novel morphological and osteometric methods for sex estimation but also for validating previously published methods. It is especially important when we are developing any kind of quantitative method that will provide precise data on probability and accuracy of classification. In these cases, our “input data” on the sex of a person should always be conclusive.

The main limitation of this study, as in all other similar studies, was the sample size. The sample in these kind of studies is always limited by preservation and availability, so we expect that discriminant functions developed in this study will be furtherly tested on the Medieval but also on the Modern sample and that their accuracy will be additionally validated. Finally, we also expect that influence of extrinsic factors on sexual dimorphism will be thoroughly studied and that in future these mechanisms will be better understood.
